# Expert considerations and consensus for using dogs to detect human SARS-CoV-2-infections

**DOI:** 10.3389/fmed.2022.1015620

**Published:** 2022-12-08

**Authors:** Sebastian Meller, Maryam Sultan Ali Al Khatri, Hamad Khatir Alhammadi, Guadalupe Álvarez, Guillaume Alvergnat, Lêucio Câmara Alves, Chris Callewaert, Charles G. B. Caraguel, Paula Carancci, Anne-Lise Chaber, Marios Charalambous, Loïc Desquilbet, Hans Ebbers, Janek Ebbers, Dominique Grandjean, Claire Guest, Hugues Guyot, Anna Hielm-Björkman, Amy Hopkins, Lothar Kreienbrock, James G. Logan, Hector Lorenzo, Rita de Cassia Carvalho Maia, Juan M. Mancilla-Tapia, Fernando O. Mardones, Leon Mutesa, Sabin Nsanzimana, Cynthia M. Otto, Marília Salgado-Caxito, Florencia de los Santos, Jeine Emanuele Santos da Silva, Esther Schalke, Clara Schoneberg, Anísio Francisco Soares, Friederike Twele, Victor Manuel Vidal-Martínez, Ariel Zapata, Natalia Zimin-Veselkoff, Holger A. Volk

**Affiliations:** ^1^Department of Small Animal Medicine & Surgery, University of Veterinary Medicine Hannover, Hanover, Germany; ^2^Dubai Police K9, Dubai, United Arab Emirates; ^3^International Operations Department, Ministry of Interior of the United Arab Emirates, Abu Dhabi, United Arab Emirates; ^4^Faculty of Veterinary Science, University of Buenos Aires, Buenos Aires, Argentina; ^5^Department of Veterinary Medicine, Federal Rural University of Pernambuco, Recife, Brazil; ^6^Center for Microbial Ecology and Technology, Department of Biotechnology, Ghent University, Ghent, Belgium; ^7^School of Animal and Veterinary Sciences, The University of Adelaide, Roseworthy, SA, Australia; ^8^École Nationale Vétérinaire d’Alfort, IMRB, Université Paris Est, Maisons-Alfort, France; ^9^Kynoscience UG, Hörstel, Germany; ^10^École Nationale Vétérinaire d’Alfort, Université Paris-Est, Maisons-Alfort, France; ^11^Medical Detection Dogs, Milton Keynes, United Kingdom; ^12^Clinical Department of Production Animals, Fundamental and Applied Research for Animals & Health Research Unit, Faculty of Veterinary Medicine, University of Liège, Liège, Belgium; ^13^Department of Equine and Small Animal Medicine, University of Helsinki, Helsinki, Finland; ^14^Department of Biometry, Epidemiology and Information Processing, University of Veterinary Medicine Hannover, Hanover, Germany; ^15^Department of Disease Control, London School of Hygiene and Tropical Medicine, London, United Kingdom; ^16^Arctech Innovation, The Cube, Dagenham, United Kingdom; ^17^Laboratory OBI K19 Biodetection Center, Hermosillo, Mexico; ^18^Escuela de Medicina Veterinaria, Facultad de Agronomía e Ingeniería Forestal and Facultad de Ciencias Biológicas y Facultad de Medicina, Pontificia Universidad Católica de Chile, Santiago, Chile; ^19^Center for Human Genetics, College of Medicine and Health Sciences, University of Rwanda, Kigali, Rwanda; ^20^Rwanda National Joint Task Force COVID-19, Kigali, Rwanda; ^21^University Teaching Hospital of Butare, Butare, Rwanda; ^22^Penn Vet Working Dog Center, Department of Clinical Sciences and Advanced Medicine, School of Veterinary Medicine, University of Pennsylvania, Philadelphia, PA, United States; ^23^Department of Animal Morphology and Physiology, Federal Rural University of Pernambuco, Recife, Brazil; ^24^Bundeswehr Medical Service Headquarters, Koblenz, Germany; ^25^Laboratorio de Parasitología y Patología Acuática, Departamento de Recursos del Mar, Centro de Investigación y de Estudios Avanzados del IPN Unidad Mérida, Mérida, Yucatán, Mexico; ^26^Center for Systems Neuroscience Hannover, Hanover, Germany

**Keywords:** canine detection, SARS-CoV-2, COVID-19, test system, pandemic control

## Introduction

**FIGURE 1:**
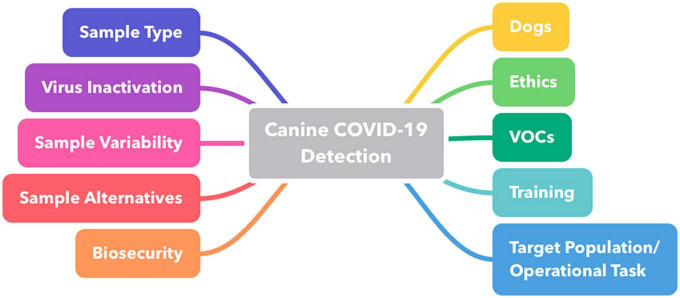
Mind map representing key areas of interest highlighted and discussed by the group of experts. VOC, volatile organic compound.

The respiratory coronavirus disease 2019 (COVID-19) caused by the severe acute respiratory syndrome coronavirus type 2 (SARS-CoV-2) quickly developed into a pandemic (1). Even though laboratory diagnostic tests and vaccines were consequently developed (2, 3), the exploration of rapidly deployable, more reliable tools for addressing the current and future pandemics was vital. Toward this goal, researchers worldwide evaluated the use of medical detection dogs as a rapid, reliable and cost-effective screening method for SARS-CoV-2 infections (4). The ability of dogs to distinguish diseases by their high-resolution sense of smell is based on the volatile organic compound (VOC)-hypothesis (5). Numerous infectious and non-infectious diseases change metabolic processes releasing characteristic VOC-patterns in the form of an “olfactory fingerprint” (6–10). Many studies have shown that dogs can detect metabolic disorders, such as cancer (11) and hypoglycemia (12), predict epileptic seizures (13, 14), or even distinguish various pathogens (8, 15–17). Approximately 78% of the 27 SARS-CoV-2-canine detection studies reviewed by Meller et al. yielded > 80% sensitivity and approximately 60% of studies yielded > 95% of specificity (4), highlighting the potential of the dog as a “diagnostic system” and its recommendation for certain settings. Despite these promising results, all studies published up to now differed in numerous design features. They were mostly designed as pilot studies and case-control selection of patients was mostly favored over a more preferable cross-sectional (“cohort”) selection [study quality assessment was conducted and presented by Meller et al. (4)]. The aim of this comprehensive review summary is to provide a general overview of the divergent aspects that may impact canine disease detection and to provide recommendations for future deployment of medical detection dogs (see also summary in Table 1). Specific emphasis is placed on the choice of dogs, training paradigms, safety aspects, sample characteristics, pre-screen processing (e.g., inactivation), and screening-population and its environment related aspects, respectively (see also Figure 1 and Supplementary Figure 1), providing an outlook and proposals for the future standardization in the use of dogs for disease detection. Ultimately, this report provides a blueprint for the potential use of medical detection dogs in future epidemics and pandemics.

**TABLE 1:** Summarizing comments and recommendations for medical canine scent detection of samples from SARS-CoV-2-infected individuals.

Disease- or metabolism-derived VOCs	Canine detection of SARS-CoV-2-infection is thought to be mainly based on the detection of volatile organic compounds (VOCs). Canine detection of VOCs can occur in real-time with high level of accuracy. However, VOC-detection is susceptible to environmental factors, which can be difficult to standardize. The success of medical detection dogs’ VOC-detection depends largely on training with the right variety of target odors.
Ethical considerations	Dogs have different personalities and experiences. They are sentient beings. The learning method should only include positive reinforcement. Dogs can fatigue and get frustrated, which should be considered in the training procedure and when they are deployed in the field. Thus, dogs require adequate work/break cycles and regular positive rewards for their work.
Dog selection	Not only anatomical but also the dog’s behavior and personality significantly impacts suitability as a detection dog. Physical and mental fitness as well as high levels of motivation are of crucial importance. Dogs should have a solid willingness to work with humans. Prior detection experience can be helpful.
Dog training	Appropriate training is the key for success in detection. Defining the correct target scent in advance is challenging, especially when the VOC-profile of interest remains unknown. The right grade of olfactory generalization vs. discrimination has to be achieved during training. Sufficient variety of new samples of symptomatic and asymptomatic patients at different stages of the disease process are here required. Duration of training can be variable and should be tailored to the individual dog’s success rate. Few days of “retraining” dogs after a longer break are sufficient to reach initial levels of detection accuracy. Line-up, scent-wheel, and detection dog training system (DDTS) have been used for training successfully. Apart from imprinting the specific scent to be recognized, also the search context needs to be trained for. While automated approaches such as DDTS might offer a more randomized and rapid training by providing higher repetition rates, line-up settings are closer to the search context in the field. Blank trials are important in order to test for forced choice decisions and to understand the individual dog’s frustration threshold. Dogs should not only be trained with negative samples, but also ideally with samples from other viral respiratory infections to reduce false-positive rates. Further work is needed to standardize and certify training procedures.
Susceptibility of dogs for SARS-CoV-2	Dogs can be infected with SARS-CoV-2, but have a low susceptibility to the virus. Clinical signs are, if at all present, mild. However, biosecurity measures for safe sample presentation, such as virus inactivation and/or safety sample containers during training and/or deployment are recommended, not only for the dogs but also for the handlers.
Sample types	Saliva, sweat, urine, and breath but also respiratory secretions and immediate body odor of SARS-CoV-2-infected individuals express specific COVID-19-associated VOC-profiles, which can be used for training and testing. Sweat collected with cotton pads is not thought to be infectious. Other sample types can be infectious and should be inactivated or presented in a container ensuring biosecurity. Only inactivation procedures should be used, which have shown not to alter the target scent and could bias canine scent detection (see below). Most studies have used sweat samples for practicality reasons. However, it is not clear if cotton-bound VOCs have a similar storage resilience than fluid-bound VOCs such as saliva or urine, which may impact training. Further work is required to provide standardized sample materials.
Virus inactivation	Beta-propiolactone (BPL), heat, ultraviolet radiation (UV), and detergent/solvent are possible measures for virus inactivation. While BPL does not appear to alter canine VOC-detection, heat and detergents might have a greater impact on altering VOC-profiles, which remains ambiguous for UV. However, the use of BPL-inactivation is more time-consuming, requiring laboratories with high safety standards. The least VOC-altering method is to omit inactivation, which works especially well for sweat samples, providing a neglectable risk for infection. In general, biosecurity aspects should never be disregarded and be approved by authorities.
Training sample alternatives	Currently, well-established sample alternatives for a more standardized training for COVID-19-detection do not exist. Artificial “VOC-cocktails,” samples from animal models, cell cultures, or pure virus protein are currently being tested and the tests are not yet conclusive. It is likely that proteins can only be used in parts of the training and that the certification procedure will require samples from SARS-CoV-2-infected individuals.
Target population and operational applicability	Studies showed high accuracies for canine COVID-19-detection within seconds with similar or better detection performances than with antigen tests. Depending on disease prevalence and characteristics of the population to be screened, the performance can alter. To ensure certainty in defining the infection/disease status of an individual, multiple back-up dogs can be involved. Changing or distracting environmental factors in the operational setting should be reduced or avoided.

## Disease- or metabolism-derived volatile organic compounds

Infectious and non-infectious diseases can produce metabolic alterations that may be associated with the release of volatile organic compounds (VOCs) from the body (6–10). In this way, specific volatile biochemical fingerprints may be detected and function as biomarkers for corresponding diseases and their clinical course, provided that appropriate sensory means are available (18, 19). The detective olfactory potential of dogs and other animals has been researched in the medical field concerning various infectious viral, bacterial, and parasitic as well as non-infectious diseases and disorders like epilepsy, diabetes, and cancer (5, 11, 20, 21). Horvath et al. demonstrated that dogs can differentiate between normal and neoplastic tissue as well as non-neoplastic disease processes such as inflammation, necrosis or emergence of metabolic products (22). For example, Ehmann et al. reported that detection dogs were able to differentiate lung cancers from chronic obstructive pulmonary disease (COPD) by sniffing the breath (23). The occurrence of specific disease-associated VOC-profiles using chemical analytical methods and technical sensory devices was shown in ovarian (24) and breast cancer (25) or in various respiratory diseases (26) and other infections (27). By applying quantitative analytical methods in animal or *in vitro* models, interesting questions about the temporal and quantitative dynamics of VOC-production across infection states and progress can be addressed. Traxler et al. (28) detected VOC-changes in the breath of pigs after influenza A infection versus control animals. Interestingly, none of the animals in the study displayed clinical signs, indicating that changes in VOCs still remain despite a lack of significant host immune responses (28). Another study measured VOCs produced by B lymphoblastoid cells following infection with specific avian and human influenza strains *in vitro*. VOCs did change depending on infection status, which coincided with the many cellular processes that occur when an organism becomes infected (29).

Gould et al. summarized that, in various viral infections, glycolysis in host cells is elevated due to the necessary energy supply for replication, accompanied with increased production of fatty acids, alkanes and related products (30). SARS-CoV-2-infections were shown to lead to characteristic immune and metabolic dysregulation in proteins and lipids in blood serum (31). SARS-CoV-2-specific biochemical processes, such as those associated with modes of entry and replication in cells, combined with induction of humoral and cellular immunologic reactions as well as the dynamic cytokine release might play an important role in COVID-19-specific VOC-expression (32).

### The smell of COVID-19

Various studies exist, which give striking insights into SARS-CoV-2-VOC-profiles with differing identifiable VOCs mainly *via* gas chromatography-mass spectrometry (GC-MS), gas chromatography-ion mobility spectrometry (GC-IMS), time-of-flight-mass spectrometry (TOF-MS) or related techniques (33–38). In principle, spectrometric techniques enable the identification and quantification of VOCs in breath samples, preceded by gas chromatographic separation if needed. Prior studies have reported quantifiable differences in about two dozen VOCs between individuals with COVID-19 versus healthy individuals as well as individuals with other respiratory diseases. Particularly striking here are COVID-19-associated elevated concentrations of certain alcohols such as butanol and propanol or derivatives (33, 35, 37, 38), aldehydes such as heptanal, octanal, and nonanal (33, 34, 36), as well as ketones such as acetone and butanone or derivatives (33, 38). Other substances with reported increased concentrations are various alkanes, alkenes, further aldehydes, aromatic substances, and their derivatives (33, 34, 36–38). Decreased VOC-concentrations in COVID-19-breath were shown for methanol (33) and – in contrast to Ruszkiewicz et al. (33) – acetone (35). In addition, Feuerherd et al. showed by headspace air sampling of virus-infected cell cultures that specific differences in 2-butanone, nonane, and pentanal concentrations represent robust discriminatory features between SARS-CoV- 2-, human coronavirus NL63-, and influenza A virus subtype H1N1-infections (39). Similarly, Steppert et al. were able to discriminate between individuals infected with influenza A virus or SARS-CoV-2 analyzing breath samples *via* IMS coupled with a multicapillary column (40). In a study from ten Hagen et al. dogs were able to discriminate supernatants of SARS-CoV-2-infected human cell cultures from 15 other viruses including coronaviridae, orthomyxoviridae, paramyxoviridae, pneumoviridae, adenovirus, and rhinovirus among others (41).

The use of electronic noses (eNoses) has also been explored by some studies for the detection of COVID-19. Sensors and nanotechnology allow to detect differences in the chemical composition of air samples by means of chemical reactions with sensor arrays consisting of specific coatings of certain metal oxides, organic polymers, nanoparticles, etc. (42, 43). The emerging differences in resistance and conductivity produce corresponding “volatile finger-” or “breathprints” *via* artificial neural networks (44). eNoses were able to discriminate breath samples between individuals with symptomatic COVID-19 versus healthy individuals (45–47) or other respiratory diseases (48), Post-COVID-19 condition (49), and non-symptomatic COVID-19 (47, 50). Two recent studies provided evidence that also dogs can detect Post-COVID-19 conditions (51, 52).

### Detection of disease-related volatile organic compounds by devices versus dogs

Despite good discriminatory potential within individual studies, the comparison of the described chemical analytical or sensor methods between studies nevertheless highlights some drawbacks of these techniques, which may create challenges for their use in an open screening process. In the following paragraphs, certain features of the canine and technical methods are critically discussed.

First, it is not ensured that all relevant VOCs are reliably detected *via* MS or sensor methods. Differences in databases and small number of metabolites available as standards complicate interpretations of MS analyses (53). Small ions, molecules or molecular fragments cannot be easily detected and make it difficult to interpret and draw conclusions about originally contained compounds. For example, small hydrocarbon-based molecules occur abundantly in exhaled breath, making their detection complicated due to overlap with molecules of similar spectra (38). In addition, certain measurable VOCs are non-specifically altered across diseases making disease discrimination prone to errors. For example, elevated propanol in breath is associated with infectious and non-infectious respiratory diseases other than COVID-19 (35, 54–57). Analogously, a certain “roughness” of detection is also given with eNoses, since the selective and susceptible coatings of the sensors might lead to physical limitations in qualitative and quantitative resolution (42, 58, 59). These aspects become impactful, especially when considering that VOCs in exhaled breath are numerous and most of the VOC-compositions have wide inter-individual variations (60). Similarly, some uncertainties exist in canine detection, as well, since research in perception and processing of certain olfactory cues in dogs is not yet very advanced. Thus, the definition of the target odor, especially in the medical field, remains one of the main challenges in canine scent detection.

Second, differences in the detection of COVID-19-VOCs across studies with MS-detection might emerge due to the choice of different detection and analytical techniques, different patient recruitment procedures and the environment (33, 37). Snitz et al. and Rodriguez-Aguilar et al., who conducted cross-sectional trials in a real-life scenario with eNoses, showed the significant impact of differing sample acquisition methods and environmental factors on the results (45, 50). Although disease discrimination was possible, certain environment-associated deterioration in eNose performance could not be excluded (50). Therefore, it is probable that the chemical analytical and sensor detection methods are susceptible to “olfactory noise” for COVID-19-detection. While these devices might feed intrinsic and extrinsic VOCs to the analyzer in an unfiltered, noisy, and “one-dimensional” manner, living biosensors such as dogs may perceive the learned sensations that are evoked due to a certain key composition, or “network,” of complex and low-concentration VOCs. Dogs are therefore possibly more capable of searching specifically for the “needle in the haystack” than current technical solutions, provided that training samples are correctly and meticulously defined according to the target condition. However, olfactory noise and other distractors may play an important role for canine detection, as well, especially when using detection dogs in the open environmental space. Further research is needed to increase control of these confounding factors.

Third, chemical analytical instruments are often stationary devices. They are mainly used offline and are coupled with software for evaluative steps. eNoses are mobile and online analysis is possible, but they require further software and deep-learning approaches in order to “learn” and analyze specific VOC-patterns. For example, the sensors must be able to detect the correct compounds and in the correct ratio and at low concentrations. The software then has to interpret the signals correctly, and environmental factors can cause difficulties, as described above. Each specific application needs considerable method development work in advance and is cost-intensive which is a drawback in rapid pandemic dynamics of emerging pathogens. Marder et al. stated that “data processing is a major bottleneck of metabolomics” (38). Furthermore, sensors often have a short life and their sensitivity deteriorates in presence of humidity (42, 48, 50). The analysis time for chemical analytical devices or sensors used for COVID-19-detection in the aforementioned studies (see section “The smell of COVID-19”) revealed a range of one to 16 min per sample. Dogs, on the other hand, are mobile and can identify COVID-19-samples within a few seconds, i.e., in real-time. This requires preceding specific canine training for high discriminating performance of approximately 4 weeks with a range of 2–15 weeks regardless of the chosen training method (when studies with dogs that had previous COVID-19-scent experience were excluded) (4). However, a variety of factors can have a large influence on learning efficiency, e.g., number of sample exposures, environmental factors, the success of odor generalization, etc. Furthermore, personality traits of dogs and emerging fatigue during work (see also section “Considerations regarding Dog Selection”) are impacting factors, which represent a disadvantage compared to well established artificial devices.

Finally, the lower limit of detection in dogs is one part per trillion (ppt), exceeding the range of detection of current available instruments by around three orders of magnitude (61–63). A new study shows that dogs are indeed able to detect even far lower concentrations, in the order of 10^–21^ (Turunen et al., unpublished). Since it was reported that VOCs from breath are released in the range of parts per billion (ppb) to ppt, dogs might appear more suitable for VOC-detection in comparison to instruments with sensitivities in the ppb range (50, 64). However, the canine range of detection was validated in controlled environments, which could mask an actual lower sensitivity. In addition, sensitivity might also depend on the qualitative characteristics of the target odor.

In hospitals and other health care facilities, chemical analytical and sensory instruments are well suited for sensitive and relatively rapid isolation of patients (33, 37), provided that they are swiftly fed with sufficient data for rapid adaptive purposes (36, 65). For external mass screening, the use of such technical devices for VOC-detection is complex due to sample processing time, limited selectivity, and increased susceptibility to material damage as well as to external olfactory noise in a poorly controllable environment. Although similar challenges may exist for dogs, their ability to learn and to process information immediately can make them more capable of searching for specific odors in real time, particularly in complex environments. However, the success of canine detection depends significantly on the training methods and the choice of the right training samples, which is one of the main challenges and disadvantages compared to established analytical and sensory methods. Finally, dogs are likely to be complimentary to sensors and analytical methods and more appropriate for certain scenarios.

## Ethical considerations for using detection dogs

One important consideration in repurposing dogs’ olfactory abilities for the detection of specific odors is that dogs are living beings with different and individual needs, characters, experiences, behaviors, and capabilities (66). In addition, these elements may differ in the same individual over time due to intrinsic and extrinsic factors. These characteristics, which from an ethical point of view must be protected and respected, considerably distinguish dogs from standardized, industrially produced test kits that have been tailored to a specific purpose (67). Ethical considerations in using dogs’ abilities for human purposes are therefore paramount. The method of operant conditioning including positive reinforcement of correct searching behavior by reward (food, toy, etc.) and absence of reward for undesired searching behavior, is considered as ethically unobjectionable, and was the method used across the canine SARS-CoV-2-detection literature (4). For the dogs, the method forms a motivation and pleasure driven detection exercise using olfaction as one of the most important sensory and cognitive tools in macrosmatics. On the other hand, one is confronted with potential short-term issues such as fatigue and/or boredom after a certain time of action, highlighting the importance of specific and individual adaptations in training and deployment according to the different personality traits of the dogs (67). However, Guest et al. drawing on their experience of deploying medical scent detection dogs, suggest that two trained dogs would have the potential to screen 300 individuals in 30 min in a COVID-19-screening scenario (47), which exceeds the capacity of current available testing methods by far. After the fast screening of a population by canine detection, reference standard reverse transcription quantitative real-time polymerase chain reaction (RT-qPCR) of positive scent detected individuals can be applied as further downstream verification (47). Such approaches were already being pursued early in the pandemic, e.g., at Dubai Airport in July 2020.

Importantly, when dogs are deployed in the field and the disease of interest has a low prevalence, reducing the opportunity for the animals to succeed in detection, positive affective and motivational states of dogs have to be sustained in order to avoid frustration (68). This can be achieved, for example, through regular rewards for the respective detection procedure or for detecting specifically prepared (positive) samples (69–71). Interestingly, variation in reward types may lead to a more pronounced maintenance of motivation in some dogs (72). In addition, adequate work/rest cycles for the dogs are of significant relevance for animal welfare and for high efficiency in scent detection work (73, 74).

## Considerations regarding dog selection

Besides a well-functioning and harmonic partnership between dog and its handler, many other individual factors can ultimately influence the effectiveness of olfactory detection. These are highlighted in the following. Three recent reviews provide a more detailed overview about the anatomy, physiology, and other factors related to canine olfaction performance (75–77).

### Intrinsic factors

#### Breed-specific anatomy and physiology

The paucity of comparative studies on the olfactory abilities of different dog breeds including intra-breed variations represents a challenge for the selection of suitable detection dogs (75, 76). Although it can be hypothesized that anatomical and physiological characteristics of the olfactory organ play a crucial role, behavioristic and mental aspects, personal traits, and experiences are of no less importance for adequate canine screening work (76).

The mechanisms involved in molecular recognition in olfactory receptors (OR) and olfactory sensory neurons and in the identification of specific odorants are still only partially understood. In this regard, the current consensus is that each OR has a characteristic ligand spectrum and each odorant can also be detected by a combination of ORs (78). Gene polymorphisms in expression of ORs in the same breed but also between breeds differ and may be used as an indicator for scent discrimination performance (79–83). In addition, the total number of neurons, i.e., the size of the olfactory epithelium, may have an effect on olfactory acuity in dogs (79), which might be due to enhanced olfactory resolution with increased numbers of neurons (84). One study showed that dolichocephalic or normocephalic dogs (often classified as scent breeds) and wolves have better olfactory capabilities than non-scent breeds and brachycephalic dogs (85). Brachycephalic breeds have less space for the olfactory epithelium to expand in the nasal cavity and less olfactory cells reducing olfactory sensitivity, and pronounced breathing issues leading to reduced cerebral oxygen supply, reduced heat elimination, and therefore to quicker fatigue (84, 86, 87). Thickened conchae and less ramifications inside the nasal cavity may be a reason for less epithelial surface (88). Brachycephalic breeds should therefore be avoided for scent detection tasks (76). Controversially and surprisingly, Hall et al. showed that pugs are able to outperform German shepherds in olfactory tasks (89), highlighting that behavioral aspects play a crucial role as well.

Furthermore, olfactory airflow in dogs in ethmoidal regions is laminar which is optimized for scent molecule transport (62, 90). This type of airflow is impacted in brachycephalic breeds due to an obstructive and deforming development of the nasal cavity and nasal conchae (87, 88), especially since the dorsal meatus in the canine nasal cavity, functioning as a bypass for olfactory laminar air supply, is only ventilated when high inspiratory pressure is applied (62, 90, 91). However, Wagner and Ruf showed that a large surface of the bony turbinates in dolichocephalic dogs is not the main reason for a better smelling ability (92), highlighting, again, the fact that breed and anatomy should not be the ultimate reason for defining a good detection dog (76).

All mentioned dog breeds in the reviewed literature of canine COVID-19-detection by Meller et al. were normocephalic breeds, which are typically used for scent detection work and are known for their outstanding olfactory capabilities and resilience (e.g., Belgian Malinois, German Shepherd, Labrador Retriever) (4).

#### Dog health, behavior, and sex

Impact of physical, behavioral, and sex related factors are less investigated than anatomical properties. In addition to a well-functioning olfactory system, a high degree of physical and mental fitness and especially motivation are essential for dogs to focus on the target scent in different environments (76, 93). Although the speed of dogs’ olfactory system is currently unsurpassable by itself, a high level of stamina, agility, athleticism, and motivation in dogs is of great benefit to enhance testing throughput. A high motivation and fitness level can compensate for difficulties in search tasks that prove fatiguing and where target odors are scarce (94–96).

Good cooperative work with humans, especially a balance between obedience and independence, is essential in deployed dogs. On the one hand, independence ensures self-determined searching strategies rather than being potentially misguided by the dog handler. On the other hand, obedience leads to efficiency, where the dog handler can narrow down the area for the search (76).

Aggression toward humans and other animals should be excluded and distractibility and anxiety levels should be as low as possible. Female dogs are generally considered less aggressive and more cooperative (97–99). In terms of neurophysiology, cells in the olfactory bulb of female dogs were shown to be more active than in males. Furthermore, female dogs have a better long-term memory (100). Neutering was assumed to decrease levels of aggressiveness and distractibility (101, 102) and males were shown to perform better in terms of directionality assessment of the target odor (103). Importantly, however, Jamieson et al. summarized that breed and sex finally should not be the crucial cornerstones to assess suitability of detection dogs since training, socialization, experience, and long-term and short-term environmental exposure can have far-reaching influences (76). Sex aspects and potential differences in terms of olfactory performance were not relevant in the screened COVID-19-detection studies (4).

Physical health of deployed dogs should always be guaranteed in the first place from the perspective of ethics and animal welfare and, secondly, not to interfere with their scent performance. Diseases and disorders capable of impacting canine olfaction are, e.g., tumors and injuries in the nasal cavity, infections like aspergillosis, distemper and parainfluenza as well as endocrinological disorders like hyperadrenocorticism, hypothyroidism, and diabetes (104, 105). Also, the function of the vomeronasal organ, supposed to be responsible for detection of pheromones and low-volatile substances, can be impacted by diseases (77). A parotitis was shown to decrease the accuracy of SARS-CoV-2-detection in one study dog (69).

SARS-CoV-2-infections can affect olfaction in people (106) and in some animal models [e.g., hamster (107) and mouse (108)]. Although dogs can be infected by SARS-CoV-2 (109, 110), typically, no clinical signs or mild and reversible signs are observed and susceptibility appears to be low (111). However, biosafety measures should be used when deploying scent detection dog teams (see also sections “Susceptibility of dogs for SARS-CoV-2” and “No viral inactivation”).

#### Dog mental condition and age

Olfaction is affected by aging processes, and can manifest in atrophic degeneration of the olfactory epithelium, decreased neurogenesis and loss of olfactory cells and their cilia (112). Wells and Hepper showed that younger dogs perform better in olfactory directionality perception than older dogs (103). In addition, pathological aging (e.g., canine cognitive dysfunction) is associated with lower olfactory capabilities (113–115), analogous to human patients with Alzheimer’s disease (116). In 27 studies reviewed by Meller et al. (4) the median age of dogs involved in COVID-19-detection was 3 years (range 0.5–12.0). Age dependent canine olfactory performance in COVID-19-detection cannot be provided as such comparisons were not in the scope of the reviewed studies (4).

### Extrinsic factors

#### Prior scent detection experience

Previous detection experience in dogs is of great advantage. However, inexperienced dogs can be trained and deployed for COVID-19-detection, as well, achieving high diagnostic accuracies (4). For example, Chaber et al. showed that inexperienced dogs were as efficient and accurate as experienced dogs (117). Experienced dogs that are accustomed to the mechanics and environment of odor detection tasks, may only need to learn the new target odor-profile, whereas more time must be allowed for inexperienced dogs to learn to handle the procedure in the setting confidently and efficiently. However, more time should be calculated when changes in setting between training and testing occur (71, 118), even for experienced dogs, in order to ensure understanding of the new search context. Interestingly, the experience of dogs in detecting odors seems to be positively correlated with the ability to cope with more complicated odor information and with stability of long-term memory (100). Inexperienced dogs seem to use olfaction to a lesser extent than experienced dogs (119), highlighting that olfaction is subject to learning processes and plasticity and can be shaped accordingly. Therefore, frequent olfactory exercises with alternating scenarios are beneficial to the training repertoire and experience.

#### Dog operational environment

High environmental humidity seems to be favorable for scent perception in dogs probably due to increased nasal humidity and enhanced odorant trapping (120), while high temperatures might have a negative impact on the general work flow (121). The dehydration of the mucosal layer in the dog’s nose can decrease the odor detection capabilities (122). The training and sample assessment by the dogs is preferably done in a spacious and conditioned room with controlled temperature and humidity. Still, dogs should be let out in between runs to avoid boredom and increase their odor detection capacity. It was found that dogs had a lower performance when they were exposed to direct sunlight and at higher temperatures (Callewaert et al., in preparation). Kokocińska-Kusiak et al. described environmental factors concerning canine scent detection in the open field, highlighting how sudden environmental changes might impact olfactory abilities (77). However, even in a more spatially restricted searching context, where dogs are involved as screening tools, alterations of external factors should be kept to a minimum. In the study of Vesga et al., COVID-19-detection dogs directly sniffing people in public transport performed at 69% sensitivity. However, those dogs had no training for 2.5 months prior to this testing scenario and still performed well (118). In the study of ten Hagen et al., dogs had high detection accuracy in line-up screenings at concerts (sweat samples, sensitivity 82%, specificity 100%) following the training phase of one to two weeks in a line-up setting (71). Apparently, dogs’ performance was not affected by potentially constantly changing odor-profiles at the testing location, although testing was restricted to a dedicated, roofed, and protected area (71). It may be probable, that a setting of stationary and somewhat isolated dog detection procedures in the form of a checkpoint, e.g., in an isolated roofed space, is more suitable, efficient, and more constant than letting dogs pass through crowds of individuals where olfactory and further environmental sensory distractions may have a higher impact (69, 123). Interestingly, there are also specific and rigorous training programs for explosive detection dogs (e.g., Vapor Wake^®^ dogs) designed for the reliable detection of odorants in aerodynamic wakes of moving individuals in crowds of people (124).

Other external influences on canine olfaction performance can originate from food and drugs. Certain food compositions and ingredients can enhance or decrease olfactory acuity (120), which seems to be dependent on the level of physical exercise in dogs. Angle et al. found benefits to olfactory performance when corn oil supplemented diets were used together with exercise (125), whereas feeding coconut oil supplemented diets without exercise impaired olfaction (126). Interestingly, relatively few studies exist concerning commonly used drugs in dogs and their impact on olfactory performance (77). Especially, metronidazole (127) and steroids like dexamethasone or hydrocortisone (128) have the potential to impair olfaction.

## Considerations regarding dog training

Training is the most critical step in predicting the success of dogs in any form of detection work. Dogs without prior odor detection training must learn the value of odor detection, associating a reward with the smell of the target sample. The physical mechanics of searching for and responding to odor in the training and testing environment may be novel to dogs, even to those with prior odor detection experience. Many dogs trained in odor detection (e.g., explosives, narcotics) are trained to recognize an odor, but are not required to discriminate between two very similar odors (i.e., human scent from a diseased state versus human scent from a non-diseased state). Therefore, dogs must learn that the background scent (i.e., individual people) can vary greatly, but the target is the common scent present in only diseased individuals, a task which requires generalization (129). Thus, defining the correct target scent in advance is crucial for the training and subsequent testing in the field (see section “Variability of samples”). Because little is yet known about the COVID-19-odor, target scent definition may seem inconsistent, especially early in a pandemic. Nevertheless, the majority of dogs involved in COVID-19-screening studies performed with high diagnostic accuracies with novel samples in the diagnostic test evaluations (DTEs) (4).

The training method used across COVID-19-studies was operant conditioning with positive reinforcement of correct searching and indication behavior using reward (food, toy, etc.) and the classical conditioning for odor imprinting (presentation and conditioning of the target scent). This method allows for an intrinsically arising motivational boost, which is the determining factor for successful learning. However, training protocols differ depending on the materials, settings, and learning approaches that were used (4). Therefore, there is a lack of standardization of canine training methods for disease recognition, especially for COVID-19, resulting in uncertainty in intra- and inter-dog reproducibility and in translation to real-world scenarios (130). Currently, standardization methods are being developed and a detailed training protocol is provided as supplemental material by Chaber et al. (117). Furthermore, ten Hagen et al. emphasized that integrating other, similarly acting pathogens into training procedures is reasonable in order to decrease the false positive rate and to sharpen the accuracy of dogs for SARS-CoV-2-detection (41). Once dogs learn to reject samples of similar pathogens that appear frequently in a population, sharper discrimination between these pathogens and the target pathogen can be achieved (41).

### Olfactory generalization

A key component for consideration during the training process is the scent generalization, which ensures that the dog searches for the common scent-profile of a target condition among all samples of interest rather than recognizing individuals (129). The degree to which generalization is required also depends on the search context. When deploying dogs as a pandemic countermeasure, exposing the dogs to numerous and varied samples from both affected and unaffected individuals will likely lead to higher proportions of correct decisions in an open field screening-scenario, where sources of olfactory confounding factors may be numerous (e.g., age, physiological condition, other diseases, diets, hygiene, habits, environment, etc.). However, too broad of a generalization gradient can also lead to a “dilution effect” of the target scent perception. In this condition, a wide range of different odors is present in the learning repertoire, which differ gradually from the target odor. Thus, too much generalization may mean that dogs also recognize odor-profiles that are merely COVID-19- or SARS-CoV-2-associated. This would lead to an increased false-positive screening rate. Training on a narrow and invariable scent repertoire, on the other hand, can lead to increased discrimination and to confident recognition of very explicit odor patterns or individual samples. This situation can be a problem for screening of a disease-associated odor-profile among plethora of individual odors, potentially leading to an increased false-negative screening rate (129). The main challenge in canine medical scent detection is to assess the origin of the olfactory profile of interest through a myriad of metabolic and other processes, and thereby to define the target odor. The lack of knowledge about the exact odor-profile of COVID-19 and whether this odor-profile is consistent among individuals, represents a “black box” for dog training and makes balanced generalization very challenging. Both balanced generalization and discrimination can be useful, depending on the search context, to enable multi-layered searches, e.g., starting with a broad screening by dog x (e.g., condition) followed by a specific search for a particular target (e.g., pathogen or variant) by dog y (see also section “Standardized sample alternatives”). In order to assess adequate degrees of generalization dogs should be regularly confronted with new samples, both during training and, more importantly, when DTE is conducted. Dogs’ reaction should always be carefully observed, especially when confronted with novel samples, to determine whether generalization processes took place. However, the exact mechanisms of olfactory generalization remain poorly understood (129). An interesting contribution could be made by studies that titrate the intensity of generalization upon detection of disease odor against dogs’ performance. In this way, rates of correct choices for defined samples, or a defined condition, could be compared between dogs trained with different odor-profiles varying in their odorant spectrum. This could provide important conclusions about the relative generalization process. However, it is probable that generalization depends on further properties of odorants, e.g., source, quality, and quantity of odorants, interactions between odorants, etc., as well as on the individual dog’s personality or learning style.

### Training duration

Training periods varied between canine COVID-19-detection studies as durations were chosen arbitrarily. Overall, dogs were trained in 2–15 weeks (median 4 weeks), including habituation (e.g., familiarization with scent work, search contexts, and workflow) and/or imprinting, for the detection of COVID-19- or SARS-CoV-2-infections, if no prior COVID-19-scent experience was present. No systematic testing for detection accuracy after different previously defined training periods has been reported and typically the increasing training performance over time was used as a basis for the decision to start the DTE (4). Vesga et al. showed that dogs still performed in an acceptable way (69% sensitivity, 94% specificity) after a training gap of 2.5 months (118). Interestingly, half a week of robust “retraining” of dogs with previous COVID-19-detection experience resulted in comparable high COVID-19-detecting performance as observed after initial training (41, 52, 71). In contrast to the results in the study of Vesga et al. (118), a recently published study highlighted that dogs indeed can remember at least 40 different defined odors, not experienced within 12 months, with 100% accuracy (131). However, the metabolism-induced smell of a disease may be more complex and more difficult to detect (and to remember) than more simple odors, especially among numerous individuals. In addition, it appears important to regularly confront dogs with fresh samples in order to react dynamically to changing disease conditions (e.g., new virus variants) early and reliably. Further research is necessary to assess the potential of canine olfactory memory in order to establish efficient training and break plans for the maintenance of high olfactory performance and for the reduction of fatigue- and boredom-related performance losses.

### Training setting

**FIGURE 2:**
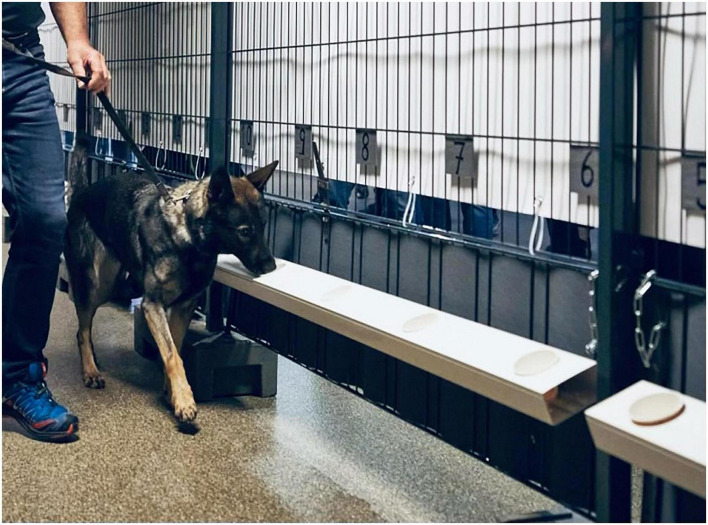
Dog and its handler working in a line-up setting.

**FIGURE 3:**
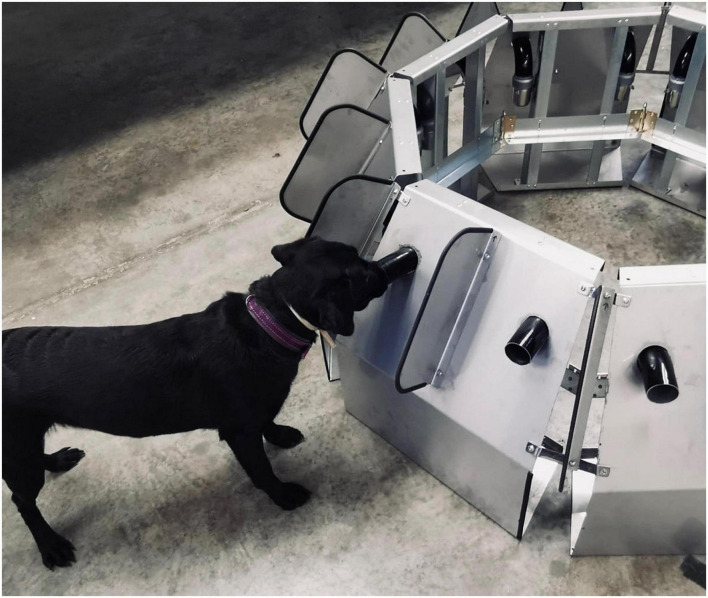
Dog working at a scent-wheel.

**FIGURE 4:**
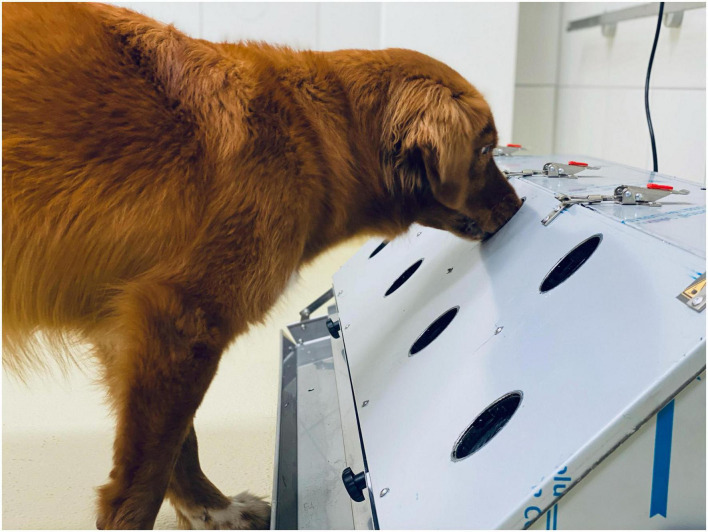
Dog working at an automated detection dog training system (DDTS).

The majority of the 27 reviewed studies by Meller et al. (4) used training with line-ups (19 studies; Figure 2). Scent-wheel training (Figure 3) was used in three studies while five studies used the Detection Dog Training System (DDTS; Figure 4), a device dedicated to the automated, randomized and software-driven presentation of samples (4). In the more classical training methods, great care needs to be taken when exchanging the scent containers, so that sequencing is randomized and blinding of the study is guaranteed which, in addition, requires sufficient personnel and material. Manual and frequent exchange of containers is time-consuming and contamination of containers needs to be avoided. In case of reusable sample containers cleaning after usage and between sessions with different dogs is of crucial importance. While in the traditional approach the dog usually works together with its handler, in the DDTS-approach the dog works independently, significantly decreasing handler bias [“Clever-Hans”-effect (132)], and the sample presentation frequency is high with multiple presentations per minute and an automated reward system. The studies that used DDTS observed generalization quickly despite a limited number of samples used (41, 52, 71, 133, 134). While such automated approaches might enable fast scent conditioning (135), they lack the reference to real-life scenarios where samples (or individuals) would be presented along a line (e.g., airports, schools, events, etc.) to the dog and its handler. Therefore, it is recommended to use a mixed approach using automated methods for initial fast, unbiased scent imprinting and generalization with subsequent habituation and training at line-up or scent-wheel settings to train dogs for systematic and controlled screening in real-life scenarios. It is also recommended to regularly challenge dogs in training with “blank trials”. These trials, which do not contain samples with the target odor, are conducted to evaluate whether dogs perform forced choice decisions, possibly due to rapid frustration after not finding the target odor. Especially, when prevalence of a certain disease is low, such frustration thresholds in detection dogs must be high and monitored. Based on the frustration level of the individual dog, target scent samples should be presented at a dog specific interval to keep frustration levels low (see also section “Ethical Considerations for Using Detection Dogs”). Of the 27 reviewed studies by Meller et al., only nine studies reported the use of blank trials (4).

## Susceptibility of dogs for SARS-CoV-2

To date, little is known about the susceptibility of dogs to SARS-CoV-2 and the disease caused by it but initial findings indicate that the susceptibility is low (111). However, studies have shown that dogs can be infected, accompanied by seroconversion, but usually do not show symptoms of disease (110, 136–140). However, a rare association between SARS-CoV-2-infections and the development of myocarditis has been suggested (141). Virus shedding seems to occur to a small extent due to limited titers and during a very short period of time (136, 139, 140). In addition, infection appears to be complicated because only a small percentage of dogs living in households of COVID-19 patients become infected (110, 137, 138). Therefore, there is currently no evidence that dogs play a determining role in virus circulation or transmission to humans, but this should not be ruled out at this stage (110). In contrast to dogs, ferrets and cats seem to be more susceptible to SARS-CoV-2-infections (111, 142, 143).

In the reviewed studies of canine COVID-19-detection, there are no reports of SARS-CoV-2-infections in the involved dogs (4). Of the three studies that conducted PCR-testing of the dogs after the tasks, none of the dogs tested positive (118, 134, 144). However, those studies had high biosafety standards. Biosafety measures should be addressed in training and testing, such as safety containers (118, 134, 145) or chemical and physical viral inactivation measures (41, 52, 71, 133, 134, 145–147). In addition, personal protective equipment should be used to protect involved individuals, even in the case that samples show low infectivity (see section “Sample types”). The following sections discuss the wide variability of sample types and inactivation measures used in reviewed canine COVID-19-detection reports (4).

## Samples for use in training and testing

Upper respiratory tract samples like nasopharyngeal (NPS) or oropharyngeal (OPS) swabs and, under certain circumstances, lower respiratory tract samples (e.g., tracheobronchial aspirates) are routinely used for the detection of viral nucleic acid *via* PCR-techniques (148). Especially RT-qPCR as well as lateral flow immunoassays (LFIA) are currently widely used to identify ongoing infections and rely on the direct detection of viral presence by identifying certain nucleic acids or antigens, respectively (149). Temporal and quantitative presence of SARS-CoV-2-RNA detected *via* RT-qPCR varies across different human biological sample types and across the duration of infection (150, 151).

Although the virus is essential for the induction of VOCs, the metabolic changes detected by dogs are not necessarily linked to the persistence of the virus, neither locally nor temporally, and VOC-release may lag or precede detectable viral infection (51, 52). A global COVID-19-VOC-profile affecting the whole organism seems to be plausible since Jendrny et al. could show that dogs were able to detect SARS-CoV-2-infections in different body fluids although being trained with only one sample type (134). How essential VOCs change over the time course, disease state, and other disease characteristics still needs to be elucidated. Nevertheless, if biological samples are used for training, it is of crucial importance to “capture” the odor-profile related to the operational usage, e.g., acute and active infections, since only then dogs can be involved as screening tools.

The following sections will give a brief overview of sample types used in the reviewed COVID-19-scent dog literature by Meller et al. (4). Sample handling and options for preserving VOCs in samples (e.g., storage, etc.) can be found in the individual study protocols.

### Sample types

#### Saliva and respiratory secretions

Most studies comparing viral content in saliva and respiratory samples showed saliva samples to contain SARS-CoV-2-RNA in patients of differing age and with differing severities of COVID-19-infections (152). Saliva may substantially contribute to the airborne/droplet transmission (153, 154). It is suggested that in the oral cavity and in epithelial cells of minor salivary gland ducts significant expression of angiotensin-converting enzyme 2 (ACE2) and transmembrane protease serine subtype 2 (TMPRSS2) may contribute to enhanced viral invasion of the host cells by coronaviruses (154–157). High levels of SARS-CoV-2 in saliva are usually already detectable at COVID-19-symptom onset, and usually the loads are similar or slightly lower than in NPS/OPS (151, 152, 158–164). Some studies showed higher viral loads in saliva in some patients or positive tested saliva samples while NPS/OPS presented negative (163, 165–167), which could be due to poor NPS/OPS sampling quality or due to earlier viral manifestation in the oral cavity (165). Nevertheless, a significant decline of viral loads in saliva takes place in the later time points of infection compared to NPS/OPS (151, 158, 159, 162, 164). Interestingly, the grade of salivary viral load does not seem to be associated with disease states (158, 168).

For scent dog detection, saliva was used in the DTEs of six of the reviewed studies, whereas upper airway samples were used in four of the 27 studies (4). Saliva is relatively easy and quick to obtain, but can contain high loads of viable SARS-CoV-2 in infected individuals. Therefore, samples have to be inactivated or presented in a high-security setting in order to protect dogs and their handlers from infection (see section “Pre-processing of samples”). These crucial steps can considerably complicate the training. Jendrny et al. showed that dogs trained with beta-propiolactone (BPL)-inactivated saliva samples can transfer their gained olfactory abilities to the detection of previously unknown non-inactivated SARS-CoV-2-positive saliva samples, and even to previously unknown non-inactivated SARS-CoV-2-positive sweat and urine samples (134). This successful transfer performance simplifies the training (and the real scenario deployment) considerably as it can be extrapolated from the results that regardless of the training samples used, COVID-19 can be detected by trained dogs in the real screening scenario based on a global and specific disease odor. Similarly, Essler et al. showed canine transfer abilities between urine and saliva as well (145). These results support the GC-MS-based studies by Penn et al. and Soini et al. who revealed that general VOC-compositions in human saliva and sweat overlap to a large extent (169, 170). The probable direct infection of the epithelial cells in the salivary gland ducts would provide a high grade of COVID-19-associated VOCs dissolved in saliva and further secretions from the oral cavity functioning as stable carrier media. However, it remains to be elucidated if the fluid-bound condition in saliva might elongate VOC-presence in contrast to non-fluid-bound VOCs as it occurs in sweat/body odor samples. Interestingly, research in biomarkers established the term “salivaomics” since composition of saliva appears to be sensitive to differing disease states of the organism (171). Therefore, it can be speculated that the metabolism-based olfactory fingerprint of COVID-19 in saliva has a relatively specific representation. Further important questions to elucidate are under what conditions and how long saliva samples can be stored without significant loss of characteristic COVID-19-VOCs, and how VOC-production and -dynamics are related to the temporal and clinical course of the infection. One of the authors successfully used frozen aliquots of BPL-inactivated saliva samples for training purposes within a year with success (personal communication).

#### Sweat and body odor

In contrast to the biological material from the respiratory tract, the potential for SARS-CoV-2-infectivity *via* sweat or skin is considered negligible. However, based on research on previously described human beta-coronaviruses, attention should be drawn to sweat as one possible vehicle of SARS-CoV-2-transmission (172). Skin, sweat and sebaceous glands express ACE2-receptors (173, 174), making SARS-CoV-2-infections of the resident cells probable (175, 176). However, viral load in epidermis and sebaceous glands was shown to be extremely low by immunohistochemical analysis (175, 177, 178). In contrast, cells in sweat glands contained high levels of viral spike proteins whereas cells in the sweat ducts contained low levels (175). Recalcati et al. tested the sweat of 22 hospitalized COVID-19 patients, the sweat of only five patients was SARS-CoV-2-positive *via* RT-PCR (179). In contrast, Arslan et al. did not detect viral nucleic acids in multiple sweat samples from both axilla and forehead in 50 patients with COVID-19 (180). Similarly, Fathizadeh et al. did not detect SARS-CoV-2 in sweat from the forehead of 25 patients with COVID-19 (181). These results indicate that despite potential viral presence in sweat glands, viral shedding through skin and sweat is unlikely (but not impossible), allowing for less strict security measures concerning sweat/body odor samples.

Sweat/body odor on pads, gauze, etc., or clothes was used in the DTEs of 20 of the 27 reviewed studies, while direct sniffing of live humans was conducted in only one study (4). Sweat and skin surface also appear to release VOCs which may vary depending on the internal health state of the organism (18). However, bacteria on the skin surface can also influence the metabolism of the released VOCs (6). Whether the composition of the microbiome on the skin has an impact on the COVID-19-associated VOC-profile, and whether these variations may alter scent dog acuity, needs to be elucidated. Nevertheless, body parts frequently or constantly exposed to personal care products, cosmetics, and perfumes are not ideal for sample acquisition since interactions of these products with bacteria and VOCs can occur. Furthermore, clothes intended for SARS-CoV-2-detection by scent dogs should not be washed before being presented to the dogs since important VOCs like organic acids would be destroyed by this process (182). Importantly, the non-homogeneous distribution of apocrine and eccrine glands in the skin implicates different compositions of VOCs depending on the sampled body region (183). Indeed, a varying collection period was applied depending on the body region across the reviewed COVID-19-detection studies. While a short swabbing of the crook of the arm, wrist, face or neck was sufficient for high diagnostic accuracies with sensitivities and specificities above 91% in three studies (69, 70, 134), studies that used axillary sweat or other sweat type chose a longer collection period of around 1–20 min (51, 117, 184–192) or even periods of hours in case of clothes (47, 144). However, in most cases these periods were arbitrarily chosen (4). Callewaert et al. (in preparation) found that 30 min sampling of the underarm skin yielded better canine results versus 15 min sampling.

Compared to saliva or urine sampling and processing, sampling of sweat/body odor on cotton pads or clothes represents a quicker, safer, and more feasible method without inactivation procedures and is well suited for rapid scent dog mass screening. However, it is unclear whether VOCs have a comparable half-life on solid materials such as cotton pads compared to liquids. This could complicate the creation of a long-lasting training sample set if not stored appropriately, but this remains speculative and needs to be elucidated in future studies. For example, Gokool et al. provided preliminary evidence that the specific odor persists for months in worn cotton shirts (193). Sweat samples for COVID-19-detection were stored cooled or at room temperature for around 2 h (194), 24–72 h (70, 185–187, 189, 190, 192), 1 week (188), or even up to 6 months in triple zip-lock plastic bags (69) before being presented to a dog. In some studies, sweat samples (and clothes) were frozen and then presented to the dogs thawed after longer storage periods in order to preserve VOCs (47, 52, 117, 134, 191). Those qualitative and temporal differences in storage did not seem to impact canine performance (4). A combination of training with inactivated saliva or other liquid-bound respiratory material with a stable VOC-profile and testing with rapidly obtainable sweat samples in a real-life scenario could be an effective, safe, and sustainable learning and testing method for infectious disease testing. However, an additional challenge with fresh sweat samples during training is recommended.

#### Urine

Viable SARS-CoV-2 or its RNA was detected in urine of infected individuals in various studies (153, 195–197). Although some studies showed no detectable virus in urine (198, 199) or, at least, very low viral loads compared to respiratory samples (200), other studies suggested similar viral loads in both sample types (153). These discrepancies might suggest that urinary transmission of SARS-CoV-2 is less likely in general, but that dynamics of viral shedding *via* urine could be highly dependent on the clinical and temporal stage of the disease (153, 200–203). These concepts are supported by a longitudinal study from Joukar et al. (204) who showed that at clinic admission of COVID-19 patients (*n* = 100), only 7% of the urinary RT-PCR-tests were positive. The maximal duration of viral persistence in urine was 11 days post admission which was shorter than for all other examined sample types (204). Similarly, Yoon et al. revealed a rapid decline of viral loads in urine to levels below detection limit after only 3 days post admission (151), narrowing the temporal window of virus detection in urine (202). Possible enhanced viral infections of the urogenital tract are plausible due to a prominent expression of ACE2 and TMPRSS2 (205) and renal abnormalities due to SARS-CoV-2-infections cannot be excluded (206, 207).

Urine was used in the DTEs of three studies (52, 134, 145) of the reviewed COVID-19-scent dog detection studies by Meller et al. (4). Chemical analyses of VOCs in urine have been used to detect olfactory fingerprints of different types of cancer (208) and bacterial infections like tuberculosis (209, 210). Since urine contains the intermediate or end products of numerous converging metabolic pathways it can be considered a VOC-rich body fluid (6), although saliva contains a larger variety of VOCs (208). However, due to glomerular filtration VOCs might be more concentrated in the urine than in other body fluids (211). Interestingly, in a direct comparison between saliva, sweat and urine, dogs were able to detect urine from COVID-19 patients with high certainty (median sensitivity 96% and specificity 98%), although the dogs had been trained with saliva beforehand. This might indicate a high concentration of COVID-19-associated VOCs in urine (134). Urine sampling requires more infrastructure, time, and effort and, therefore, is less suitable for mass screening scenarios compared to saliva or sweat. In addition, viral inactivation or high-security measurements should be considered due to the potential risk of viral transmission. Due to the high detection accuracy achieved in the study from Jendrny et al. (134), testing of urine could be used for additional *post hoc* confirmation after detection of a positive case during screening with other sample types. As with other sample types, however, optimized storage properties still need to be investigated. Furthermore, aspects like diet can impact urinary VOCs significantly (6), which needs to be addressed in future studies.

#### Breath

Exhaled breath contains high concentrations of various particles and molecules (212–214) including VOCs (60), with differing compositions among certain pathological conditions (6, 215). Although “violent” expiratory events such as coughing and sneezing have previously been considered the main contributors to infectious aerosol and droplet infections (216), aerosols generated by breathing can transmit SARS-CoV-2 and may have a major impact on the infection dynamics (217, 218). Breath VOCs were already investigated in many other diseases (19, 219) and initial approaches have been made in COVID-19 (see also section “The smell of COVID-19”).

Breath samples were used in the DTEs of six of the COVID-19-detecting dog studies, especially in combination with masks (4). The collection and conservation of VOCs from breath is challenging. Lomonaco et al. showed that general VOCs of breath samples stored in sorbent tubes at room temperature were stable up to 72 h (220). A study by Kang and Thomas stated that significant loss in some endogenous breath VOCs was already discernible after 6 months of –80°C storage, although specialized adsorbent tubes were used (221). It is therefore probable that VOCs in masks, similar to cotton pads or clothes, have a storage resilience of shorter duration. However, Guest et al. showed that clothes (socks) gave a stronger specific olfactory signature for dogs than breath samples (face masks) (47). This suggests a high inter-individual VOC-variability in breath samples (60) (see also section “Detection of disease-related VOCs by devices versus dogs”). Furthermore, higher storage temperatures drive a greater loss of breath VOCs on adsorbent materials (222) and longer storage times can lead to exogenous contamination (223). These properties impair the establishment of stable breath sample sets for training. On the other side, subtle skin abrasions in masks, cotton pads, and clothes certainly contribute to a prolonged retention of certain VOC-profiles (see also section “Sweat and body odor”) (6). Due to the impressive acuity of canine olfaction, the potential storage artifacts of breath samples might represent a negligible drawback, this issue however has to be addressed in further studies.

Unlike eNoses, into which the breath sample is usually fed directly, the direct presentation of pure breath in training and in real-life screening to the dogs is challenging, which is not the case for solid-/adsorbent- or fluid-bound biological material. Furthermore, due to technical and hygienic reasons, the throughput rate of current eNoses in real-life screening is lower (minutes per sample) than the throughput rate of trained dogs evaluating line-ups with self-taken sweat samples (seconds per sample) (69, 71) (see also sections “Detection of disease-related VOCs by devices versus dogs” and “Sweat and body odor”). In terms of breath VOCs, masks (or specialized adsorbent material) would be more suitable than pure exhaled breath both for canine training and screening. However, generating those mask samples generally required a longer duration of approximately 10 min to 24 h with a median of 180 min (47, 144, 146, 147, 190). Vlachová et al. however, conducted breath sampling on sterile surgical compresses of only 3 min (189). Furthermore, due to the evidence of airborne/droplet infections for SARS-CoV-2, biosecurity associated with the immediate presentation of pure breath samples is more complex than presentation of carrier material-bound samples.

#### Variability of samples

Origin of samples for training and DTE purposes is an important factor due to the potential contaminating impact of environmental VOCs (6, 208, 215, 224). For example, it could be a major issue if samples from SARS-CoV-2-positive patients originated from only one facility, conditioning dogs on facility-associated smell rather than SARS-CoV-2-associated smell. Likewise, if SARS-CoV-2-negative patients are collected from a different environment, e.g., the community, and SARS-CoV-2-positive patients are all collected from hospital environments, the systematic difference between the samples may lead to inaccurate responses by the dogs.

Similarly, geographical conditions may also have an impact on VOC-profiles (225). Chaber et al. showed slight differences in canine olfactory performance depending on the geographical origin of samples (117). However, special care should be taken to ensure that handling of utensils during sampling and processing proceeds in the same manner for both negative and positive samples across testing locations (47, 226, 227). In addition, Callewaert et al. (in preparation) found that dissimilarities in canine performance occur depending on the carrier material (e.g., cotton pad, cotton gauze, commercial odor carrier, etc.). Therefore, it is advised to use one and the same carrier throughout training and DTE.

For training and DTE purposes, a large representation of different demographic aspects (sex, age, etc.), localities of sample origin, and temporally different stages of infection among SARS-CoV-2-positive and -negative samples is crucial to adequately map the olfactory fingerprint of the disease. In addition, other aspects such as pre-existing infectious and non-infectious pathological conditions, recovered SARS-CoV-2-infections, Post-COVID-19 condition, COVID-19-vaccination status, or differing virus variants may play an important role for VOC-patterns and are subject of current research (51, 52, 69, 71). ten Hagen et al. studied the ability of dogs to discriminate between SARS-CoV-2-infections and other viral respiratory infections in NPS/OPS and infected cell cultures, when trained with saliva from SARS-CoV-2-positive individuals or with SARS-CoV-2-infected cell culture supernatants. Although sensitivity was lower (61.2–75.8%) than in other studies from the same laboratory, dogs rejected the samples of other viral infections in the DTE more often than SARS-CoV-2-infected samples, which is reflected by a high specificity of 90.2–95.1%. This indicates that further respiratory viral diseases defined as SARS-CoV-2-negative samples should always be integrated into training procedures in order to enhance diagnostic acuity for SARS-CoV-2 (41).

In terms of infection state, the crucial intervals dogs should be able to recognize is any phase in which viable virus is shed in order to contain the pandemic effectively. Therefore, samples across all phases of infection should be used for training in order to reliably indicate all potentially changing relevant odor-profiles in the course of infection. However, further research is needed to evaluate if and how dogs are able to transfer their olfactory detection abilities from a certain stage of disease to another. Recent studies found that dogs which were trained with samples from acute SARS-CoV-2-infection did not indicate patients with Post-COVID-19 condition as positive, when tested versus acute infection. Nevertheless, when tested against samples from healthy individuals, Post-COVID-19 condition samples were identified (51, 52). These results might suggest a titration effect, which could be based on a slow gradual decomposition of characteristic VOCs even if the virus is only residually or not present anymore.

Furthermore, an appropriate mapping of disease severity (e.g., asymptomatic, mild, severe) should be taken into account and integrated into training. However, COVID-19-VOC-measurement indicated that there was no relationship between VOCs and viral loads (34) or disease states, although mainly severe cases were included (36). Importantly, more research is needed to explore to what extent PCR-cycle threshold values, representing viral loads, influence canine olfactory performance [see also (47)].

Dogs can even be trained to certain concentration differences of the target scent (228). For example, this is used in diabetes alert dogs, which detect increases or decreases of blood glucose values of patients beyond predetermined levels (229). This emphasizes that the samples used in canine training procedures must be as versatile as possible. The issues described in this section will be minimized when further efforts are made in the profiling of critical SARS-CoV-2-VOCs and in the processing techniques of samples in order to reduce olfactory noise from potential exogenous and irrelevant endogenous factors (215). A crucial question which arises is whether training conditions can be reduced to the lowest common denominator by, for example, training with pure viral proteins or proteins produced in cell cultures or animal models (see section “Standardized sample alternatives”). Cell cultures were used in one (41) of the 27 reviewed studies by Meller et al. (4).

### Pre-processing of samples

Many protocols for inactivation of viral pathogens with differing grades of loss of functional and structural viral integrity exist. The main purpose of viral inactivation in scent dog detection studies is the safe handling of training samples for animals and humans. On the other side, olfactory fingerprints of samples deriving from SARS-CoV-2-infections have to be preserved, probably requiring gentle inactivation methods. Different approaches up to renunciation of inactivation procedures were used in the reviewed COVID-19-scent dog literature (4), which is discussed below. The study from Jendrny et al. revealed that inactivated samples can be used for training to subsequently screen non-inactivated “armed” samples with a median sensitivity and specificity of 84 and 95%, respectively (134).

#### Beta-propiolactone

Beta-propiolactone (BPL) is an organic chemical compound which has historically been used for effective inactivation of various known viruses (230), especially in the field of vaccine development (231–234). BPL inactivates SARS-CoV-2 as well (235). Its inactivating properties are based on opening its lactone ring which is unstable in aqueous media and highly reactive (230). Due to rapid hydrolyzation in aqueous media, the substance is transformed within a few hours to non-toxic 3-hydroxypropionic acid making it highly suitable and safe for biological preparations (236). Despite the rapid degradation, viral activity has usually subsided long before the last detectable residuals of BPL in samples have been measured (230). BPL appears to have affinity for viral nucleic acids blocking viral replication while mostly sparing the protein structures, which preserves the immunogenicity of the virus. However, not all the organic chemical modifications coming from BPL are elucidated and proteins may be affected as well (237). On the other hand, Determann and Joachim showed that a higher reactivity toward certain functional groups of amino acids results from a lower hydrolyzation capacity of the medium (238), highlighting that water is a preferred nucleophilic reagent of BPL. In summary, variations in nucleophilic characteristics of the reaction with BPL and the “nucleophilic potential” as well as further physicochemical properties of the medium might explain why varying quantitative and qualitative dynamics among reaction products from different organic compounds exist (237). In the study from Jendrny et al. dogs did not smell a relevant difference between BPL-inactivated (training) and non-inactivated (DTE) SARS-CoV-2-infected samples (134). Although more research is needed in this field, this might indicate that nucleophilicity of relevant VOCs is low and that microenvironmental aspects of the samples could further contribute to the lack of involvement of respective VOCs in the reaction with BPL so that those are kept preserved. Furthermore, it is possible that the BPL-manipulation has no effect on the high discriminatory power of the dog’s olfactory system. It is noteworthy that in the first work by Jendrny et al., non-inactivated negative samples were used in addition to BPL-inactivated negative samples (133). The dogs did not indicate the latter more often than the former even though they were trained with BPL-inactivated positive samples (133). Only three of the reviewed studies used BPL for viral inactivation in their DTEs (41, 52, 133). However, in terms of safety versus VOC-preservation, BPL inactivation represents a highly effective and reasonable method.

#### Heat

Heat inactivation is a possible and common method to destroy viral pathogens effectively (239, 240). At the same time, maintenance of antigen integrity is important to preserve the diagnostic value of samples, e.g., for serological analysis (240–247). Heating methods can prevent infectivity of SARS-CoV-2 and at the same time preserve RNA when appropriate temperatures are applied (247). In contrast, BPL preserves proteins but not RNA (see above). Heat has denaturizing properties on proteins and other compounds leading to disruption in the interaction between virion and cell. Even slight alterations might also have a crucial and persistent impact on quality of the VOC-emitting properties of organic material, changing VOC-concentrations and their chemical composition. In addition, Lomonaco et al. showed that heat treatment is able to alter VOC-composition in human breath samples (220).

Heat inactivation or treatment in the DTEs was used in three (118, 145, 147) of the reviewed studies (4). Essler et al. (145) trained dogs with detergent-inactivated urine (see below) and tested the dogs for detection of heat-inactivated urine. Especially when dogs were confronted with a novel heat-inactivated sample, overall sensitivity was only 62%, whereas specificity was 98%. This may indicate that – at least in relation to detergent treatment – heat may alter critical COVID-19-VOC-profiles to a certain extent inducing uncertainty, or that the use of detergent inactivation made the odor more obvious. However, it has to be mentioned that these transfer trials consisted of only one set of presented samples to eight dogs. Interestingly, the performance of dogs, which were trained with heat-inactivated samples and tested with new heat-inactivated samples, deteriorated significantly, which possibly was due to a poor generalization process as sample availability was limited at the time the experiments were performed (145). Possibly, the process of heat-inactivation might produce different VOC-profiles among individual samples, depending on their original chemical and physical composition. The learned VOC-spectrum would thus present too broad to be finally used in detection of COVID-19-specific smell with adequate generalization and high diagnostic acuity. The assumption of global and individual changes in the key VOC-profile through heat-inactivation is also supported by the fact that the olfactory transfer performance from heat-inactivated urine-training to heat-inactivated saliva-testing produced very low sensitivities in two trials (11 and 22%, respectively), whereas the accurate recognition of negative samples was maintained (specificity of 94 and 100%, respectively) (145). However, only one positive sample was presented per trial across nine dogs. Furthermore, the discussed aspects of heat treatment remain speculative since other possible complicating factors have to be taken into account. In contrast, Jendrny et al. showed that dogs’ transfer performance from BPL inactivated training samples to completely novel non-inactivated samples of the same and even different type is maintained at the same or even higher levels (134). BPL seems to retain the assumed global COVID-19-associated smell. Heat-inactivation appears to be more time-saving and cheaper than BPL-inactivation, but the former might lead to less robust learning results in dogs. Consistent with those statements, Salgirli et al. have also reported that dogs initially had problems recognizing heat-inactivated masks worn by COVID-19 patients when previously trained with non-inactivated masks (147). In contrast, Vesga et al., who used heat treatment in order to prevent proliferation of microbiota, reported a high performance quality of dogs, however, the treatment was not further specified (118).

#### Ultraviolet radiation

Ultraviolet-C (UV-C) radiation may be used to inactivate coronaviruses effectively (240, 248–250). It acts mainly by photochemical conversions of heterocyclic bases in the structure of nucleic acids without spontaneous reversion (251–253). Amino acids are affected to a lesser extent while carbohydrates and lipids are hardly modified (251). Mendel et al. used 10 minutes of UV-C-irradiation (254 nm) per side of mask material for SARS-CoV-2-positive cases, for canine training and for DTEs (146). Similarly, Salgirli et al. also used UV-inactivation (147). However, it is a significant concern of methodology in both studies that it is not clearly stated whether negative samples were also inactivated in order to control for potential pronounced or subtle UV-induced alterations in COVID-19-associated VOCs. In an additional experiment Mendel et al. showed that UV exposure did not result in statistically significant alterations in headspace solid phase microextraction GC-MS-profiles of at least 36 typical human-derived scent compounds pipetted on unused masks, suggesting a lack of significant photocatalytic effects on these VOCs (146). Conversely, UV-radiation of different wavelengths (especially UV-C) and dosage can have a great photocatalytic impact on gaseous emissions and VOCs by eliminating many of them from air samples, even within seconds (254–257), or from liquid media (258, 259). Therefore, the extent to which specific COVID-19-associated VOCs are altered by UV-irradiation remains uncertain and needs to be elucidated. If there are alterations, it has to also be clarified whether the discriminatory power of the canine olfactory system is nevertheless sufficient to compensate for those changes.

However, Mendel et al. (146) reported in two cases that trained dogs were able to indicate locations at workplaces where SARS-CoV-2-infected individuals had been situated 3–4 weeks prior to canine inspection. It would represent a promising indication that UV-irradiation might have no relevant effect on COVID-19-associated VOCs and that the temporal range of detection might extend well beyond acute infections, however, these are only few individual cases reported (146) and it remains questionable whether COVID-19-associated VOCs persist in a confined area for such a long time without appropriate storage [SARS-CoV-2 itself survives only a few days in the environment (260)]. In summary, comparative studies of training with UV-inactivated and DTEs with new, non-inactivated samples under high security standards (see also section “Susceptibility of dogs for SARS-CoV-2”) are an essential step to ultimately verify the suitability of UV-inactivation for establishing canine training sample sets. Although the actual process of viral inactivation by UV takes longer than by BPL (230), the use of UV would be a time-saving and an ecological method since, in contrast to BPL- or detergent-inactivation, no chemicals, no targeted chemical manipulations of the samples, and no waiting time for hydrolysis are required.

#### Detergent–solvent

Detergent/solvent applications are a further method for efficient viral inactivation by complete destruction of the lipid membrane of enveloped viruses while preserving the structure of proteins from the virus and from the biological microenvironment (261–263). This method is widely and commercially used especially in the treatment of therapeutic human plasma, as it robustly destroys enveloped viruses while at the same time retaining physiological activity levels of plasma proteins (262, 264). Nonidet™ NP-40 in combination with further detergents seems to successfully disrupt coronavirions (240) and was used by one canine COVID-19-detection study for urine inactivation (145). A possible VOC-alterating effect of Nonidet™ NP-40 or the closely related substance Triton X-100 on VOCs in treated samples is not elucidated. They represent gentle inactivation methods, but it might be assumed that lytic effects on membranes of contained cells (265) might slightly change the biochemical properties of those samples. Triton X-100 has a vapor pressure of 130 Pa at 20°C and is therefore considered an organic volatile substance (266). It can therefore be assumed that the detergents themselves change the odor-profile of the samples while they are still dissolved. In order to clarify these issues, comparative canine olfaction studies with both detergent-inactivated and non-inactivated positive and negative samples are necessary. However, Essler et al. (145) could show that cognitive transfer from detergent-inactivated to heat-inactivated samples is possible. Although sensitivities decreased, this may also be due to heat-inactivation [(145); see also section “Heat”]. Finally, chemical treatments with detergents or BPL are more environmentally damaging, time-consuming, and eventually more expensive than, for example, the use of UV-C. Nevertheless, they appear to allow satisfactory and safe olfactory transfer to non-inactivated samples in canine COVID-19-detection (134, 145).

#### No viral inactivation

No inactivation can represent a biosafety issue, but is probably also the best method for the preservation of crucial VOC-profiles. SARS-CoV-2 can survive a few days in the environment depending on the type of contaminated surface (260). In secreted biological material like aerosols, virus was shown to be infectious for minutes to hours (260, 267) whereas other studies show higher viral activity up to 21 days in different body fluids like e.g., sputum, saliva, urine, and blood, depending on seasonal factors (268). No virus inactivation was used by the majority of reviewed canine COVID-19-detection studies in their DTEs (*n* = 21) (4). Sweat samples were the main material used without inactivation which *per se* have no high infectivity (see also section “Sweat and body odor”). Similarly, four studies did not use inactivation of mask or clothes samples (47, 144, 147, 190). Apparently, the material on which sweat or body odor was collected impacts the viral persistence as well, since cotton and related material seems to ensure decomposition of its RNA within minutes (269, 270). The duration between sample acquisition and presentation to dogs was variable across studies between hours and months (see also section “Sweat and body odor”). However, in some reports, inactivation was omitted also in other sample types like saliva (184) and nasopharyngeal secretions (144) without use of further biosafety measures. Leaving out inactivation generated robust and good results (4) suggesting that characteristic VOCs outlast the virus presence or at least high viral loads. Nevertheless, independent of inactivation status, special safety measures should be used (118, 134, 145), e.g., Training Aid Delivery Device (TADD) containers (134, 145), which are supposed to allow odor particles to pass through but not droplet- or particle-bound virions, in order to protect both animals and humans (134). This is especially interesting for training purposes where body fluids with higher viral loads may be used. For detection of other infectious diseases, corresponding data about pathogen dynamics in body fluids and environment should be used, or reliable data should be generated first in the case of future emerging zoonotic diseases in order to determine susceptibility of dogs to pathogens of interest and to guarantee adequate safety.

### Standardized sample alternatives

Training sample sets derived from naturally obtained human biological fluids can be used for extended periods of time. However, more research is needed in appropriate storage conditions (see section “Sample types”). In addition, acquiring samples is not trivial both from an ethical and logistic sense, especially early in a pandemic or while pandemic dynamics are low. Furthermore, storage duration and divergent storage conditions can affect VOC-patterns (221, 271, 272). There may also exist uncertainties regarding true infection status with possible false negative or false positive PCR-status potentially corrupting the sensitive training process for the right olfactory cue, differences in temporal and clinical infection states, demographic differences, etc.

Producing specific COVID-19-associated VOC-profiles artificially for dog training purposes represents a challenging endeavor although first approaches with a “VOC-cocktail” have been conducted in combination with eNoses (273). However, sensor array composition of the eNose and environmental influences still represent a major limitation and studies are merely scratching the surface of decoding the volatilome of SARS-CoV-2-infections (see section “The smell of COVID-19”). For the current state of the canine COVID-19-detection research, it was important to cover the majority of VOC-variations, which can emerge from varying disease-associated factors, for adequate broad training and generalization. Nevertheless, it is only the attempt to “catch” the true critical COVID-19-odor of an active infection in an as broad as possible way, for the simple reason that the critical VOC-composition is not known yet but at the same time early investigation of anti-pandemic measures appeared reasonable. In this way, dogs were taught to perceive key signals from a broad array of positive samples, which were not present in a broad array of negative samples. Therefore, dogs did not learn to detect an absolute COVID-19-VOC-profile, but a certain scent-profile of relative difference to what healthy individuals did not express.

The VOC-hypothesis is based on viable metabolic entities with the result of VOC-production, which may have fingerprint-like properties for certain pathological conditions, e.g., viral infections. Some have suggested that dogs might be able to detect viral proteins, i.e., spike proteins (51, 52), which could be perceived by olfaction without any metabolic intermediate step. Amino acids are not among the substances typically defined as odorants, and to date have been little studied in the context of odor perception, except in fish (274–278). Humans have been shown to be able to distinguish among certain amino acids by olfaction (279, 280). Whether dogs are able to smell parts of the pure SARS-CoV-2-proteins and reliably discriminate it against other distractors is currently being investigated. In this context, the function of the vomeronasal organ in dogs should be emphasized, which serves as an additional olfactory organ for intra-species communication through pheromones and is located rostrally at the bottom of the nasal cavity (5). Interestingly, in contrast to the main olfactory organ, the vomeronasal organ is capable of detecting non-volatile molecules of higher molecular weight, such as proteins (281), which might indicate the presence of different receptor cell types in both olfactory organs (282).

If dogs are able to smell viral proteins, a standardized, broad, and sustainable training infrastructure based on appropriately manufactured proteins could be established and research is already underway. Safety would be guaranteed due to the absence of the viable virus, however, the risk of contamination of such sensitive samples is high. In addition, costs of sampling body fluids versus production of protein samples must be considered. An essential consideration, however, is the periodic emergence of new variants of SARS-CoV-2 with differing mutations in spike genes and protein expression (283, 284). When dogs are trained on a single protein the spectrum of detection would be extremely narrow and certainly highly specific, increasing olfactory discrimination (129), but it has to be studied whether it would suffice to cover different virus variants. It would therefore seem reasonable to mix the variants during training sessions according to the current viral occurrence in the population. However, it may take some time before the next corresponding protein is available after discovery and identification of a new variant.

It is probable that the impact of viral variants on variation in COVID-19-VOC-patterns is less pronounced. Chaber et al. stated that dogs had no difficulties recognizing the virus despite being confronted with different strains in biological samples (117). On the contrary, Kantele et al. showed a significant difference in accuracy between variants, when the training included only biological samples with the wild-type virus (69). Furthermore, by using only proteins for training, it would be essential that viral material is present and “readily accessible” in the screening samples of diseased individuals, which is not always the case for sweat/body odor or urine as described above (see section “Sample types”). In contrast, only infected individuals who acutely excrete the virus would be detected in this way sparing individuals who do not shed the virus anymore but still express COVID-19-VOC-patterns. Nevertheless, this can be deceptive because the viral load may vary temporally across the samples while the individual is still infectious or may depend on vaccine-induced immune response (see section “Sample types”).

In order to circumvent these issues, supernatants from infected human cell cultures (41) could be used for additional training with negative/distractor samples belonging to the same culture or with different cell lines among positive and negative samples, profiting from the advantages of VOCs. The combinatorial approach of VOCs and proteins (e.g., one or more “sets” of specifically trained dogs, see section “Olfactory generalization”) could maintain high levels of sensitivity in general screening and be used in special confirmatory cases as a highly specific detection method for certain dangerous viral variants, which could be continuously updated in dogs’ olfactory memory (117).

Although cell cultures are a very interesting alternative, it should be noted that the VOC-profile does not necessarily correspond to the versatility of VOC-patterns from naturally obtained biological samples. Murarka et al. trained dogs with an ovarian cancer cell line and showed that olfactory transfer or switch from cell culture to samples of patients with ovarian cancer did not readily occur in dogs (285). Similarly, there was a lower detection ability in SARS-CoV-2-positive cell culture supernatants after dogs had been trained with naturally acquired saliva samples (41). These problems could be circumvented to some extent by using different cell lines in cell cultures, but mimicking the olfactory versatility of naturally acquired samples remains difficult. Another alternative may be the use of SARS-CoV-2-training samples from animal models. Nevertheless, the question of effective translation to human derived VOCs needs to be addressed (286). Despite the great advantages of sample alternatives with regard to trainability and standardization, the use of “real” biological samples will probably still be necessary to prepare dogs for real-life screening scenarios.

## Target and screening population and the operational applicability

The World Health Organization (WHO) and the German Paul Ehrlich Institute (PEI) recommend thresholds for diagnostic sensitivities and specificities for point-of-care-antigen tests to be more than 80% and more than 97%, respectively (287). 78% of reviewed canine detection studies showed ≥ 80% sensitivity and 60% of studies showed ≥ 95% specificity. Therefore, dogs’ detection is in line with or even better than other rapid diagnostic tests. Dogs achieved even better performances when only considering high-quality studies with a low risk of bias (4).

However, when considering the entire components influencing the dog as a detection system, it has to be taken into account that the characteristics of the population to be tested has its impact on the accuracy as well. Besides the actual prevalence of COVID-19 within the target population to be screened the detection performance differs between different populations and search scenarios, which has a direct impact on the practicality. Therefore, the calculation of expected positive and negative predictive values is crucial for the decision on screening scenarios in order to avoid any vilification of the dogs’ detection (Table 2).

**TABLE 2:** Positive and negative predictive values for dogs’ performance of 90% sensitivity and 99% specificity and for the recommendations of the World Health Organization (WHO) and Paul Ehrlich Institute (PEI) among different COVID-19 prevalences in the target population.

COVID-19 prevalence	Dogs’ performance (SEN = 0.90, SPE = 0.99)		WHO and PEI recommendations (SEN = 0.80, SPE = 0.97)	
	PPV	NPV	PPV	NPV
0.0010	0.0826	0.9999	0.0260	0.9998
0.0011	0.0902	0.9999	0.0285	0.9998
0.0012	0.0976	0.9999	0.0310	0.9998
0.0013	0.1049	0.9999	0.0335	0.9997
0.0014	0.1120	0.9999	0.0360	0.9997
0.0015	0.1191	0.9998	0.0385	0.9997
0.0016	0.1261	0.9998	0.0410	0.9997
0.0017	0.1329	0.9998	0.0434	0.9996
0.0018	0.1396	0.9998	0.0459	0.9996
0.0019	0.1463	0.9998	0.0483	0.9996
0.0020	0.1528	0.9998	0.0507	0.9996

SEN, sensitivity; SPE, specificity; PPV, positive predictive value; NPV, negative predictive value.

It is furthermore important to note that not all studies relied on a single dog’s decision to determine sensitivity and specificity. In particular, in some cross-sectional studies, decisions from multiple dogs were used to ensure certainty in defining the infection/disease-status of tested individuals (69, 71, 191, 194). Those considerations, which also might depend on the number of available trained dogs, are important especially for the planning and conduction of a screening test. Furthermore, changing and distracting environmental factors should be reduced or avoided in the operational screening setting (see also section “Dog operational environment”).

## Dog detection as the one health approach to tackle COVID-19

The increase of zoonotic infectious diseases highlights the importance of collaborative, multisectoral and interdisciplinary work to address challenges that could impact public health, animal health and production, and environmental conservation. The World Health Organization (WHO), the Food and Agriculture Organization of the United Nations (FAO), the World Organization for Animal Health (WOAH) and the United Nations Environment Programme (UNEP) have established an intersectoral collaboration aiming to implement initiatives under the concept of “One Health” to address main global problems at the human-animal-environment interface (288). The four organizations are working together to mainstream One Health so that they are better prepared to prevent, predict, detect, and respond to global health threats and promote sustainable development. The use of COVID-19-detecting dogs is a great example of using this concept to respond to the current COVID-19-pandemic. In this review, we showed how multi-sectoral communication and joint work resulted in the generation of evidence that the use of dogs trained to detect SARS-CoV-2-infections has been shown to be a rapid, mobile, and non-invasive tool for early detection of affected individuals. Collaborative efforts are crucial to minimize the rapid viral transmission requiring massive testing (289, 290). The use of detection dogs to pre-screen infections among the population could overcome the overloaded response capacity of laboratories due to the higher number of required tests, the lack of needed reagents to perform these tests, and technical issues in sampling infected individuals (i.e., inappropriate sample collection, storage, or transportation) or false-negative results related to the disease status with low viral multiplication levels (291–293).

## Conclusion

Dogs can detect samples from SARS-CoV-2-infected individuals with a high degree of diagnostic accuracy. However, the search context, study design and quality of the current studies varied considerably, and only a small percentage of studies were of high quality with a low risk of bias. In contrast to an industrially produced test kit, dogs and their olfactory performance are naturally subject to many variations. In addition, disease detection involves difficult to measure and volatile amounts of substances and little is known about the olfactory dynamics of a pathological process, making it difficult to control the process of adequate odor imprinting. However, the evidence of canine COVID-19 recognition has been replicated by several different groups, and the dog proved to be an incomparably fast detection tool. Importantly, in epi/pandemic conditions, dogs can be trained quickly with a good level of sensitivity before specific laboratory methods are available, helping with isolation of infected patients presenting with or without symptoms. Therefore, further research on influences of the odor profile (or the perception of it) by factors such as training sample number and type, sampling method, inactivation type, training procedures, dogs’ personalities, environment, translation from training to test scenario, etc. proves to be very important for harmonization and optimization of canine scent detection and for maintenance of high study quality. Those considerations pave the way for the canine olfaction to become a reliable, stable and quick test method. Thus, standardization and validation processes such as those used in the field of drug and explosive detection dogs are urgently needed, if medical detection dogs should be deployed in the field to detect samples from SARS-CoV-2-infected individuals.

We recommend the use of dogs as VOC-detectors in mass screenings as a quick, highly adaptive, and effective countermeasure both at the emergence and also in the further course of a pandemic, provided that sufficient numbers of diverse positive and negative, high quality, safe samples for training purposes can be generated early, the pathophysiological condition of those samples is known with a high certainty, and that training procedures, dogs, and their handlers are certified similarly as described for scent detection in explosives (294).

## Author contributions

HV and SM discussed and planned the form of the comprehensive analysis. SM wrote the manuscript and conducted the main literature research. All other authors have contributed significantly to the completion and development of the review and provided their expert opinion. All authors have read and approved the final manuscript.

## Supplementary material

Supplementary Figure 1 Detailed mind map representing areas of interest and related aspects that played a role in the reviewed canine COVID-19-detection studies by Meller et al. (4) and have been highlighted by the experts in this publication.
