# Supplementary figures and images for: Expert considerations and consensus for using dogs to detect human SARS-CoV-2-infections
Source: Front Med (Lausanne). 2022 Dec 8;9:1015620. doi: 10.3389/fmed.2022.1015620 (PMC9773891; doi:10.3389/fmed.2022.1015620)

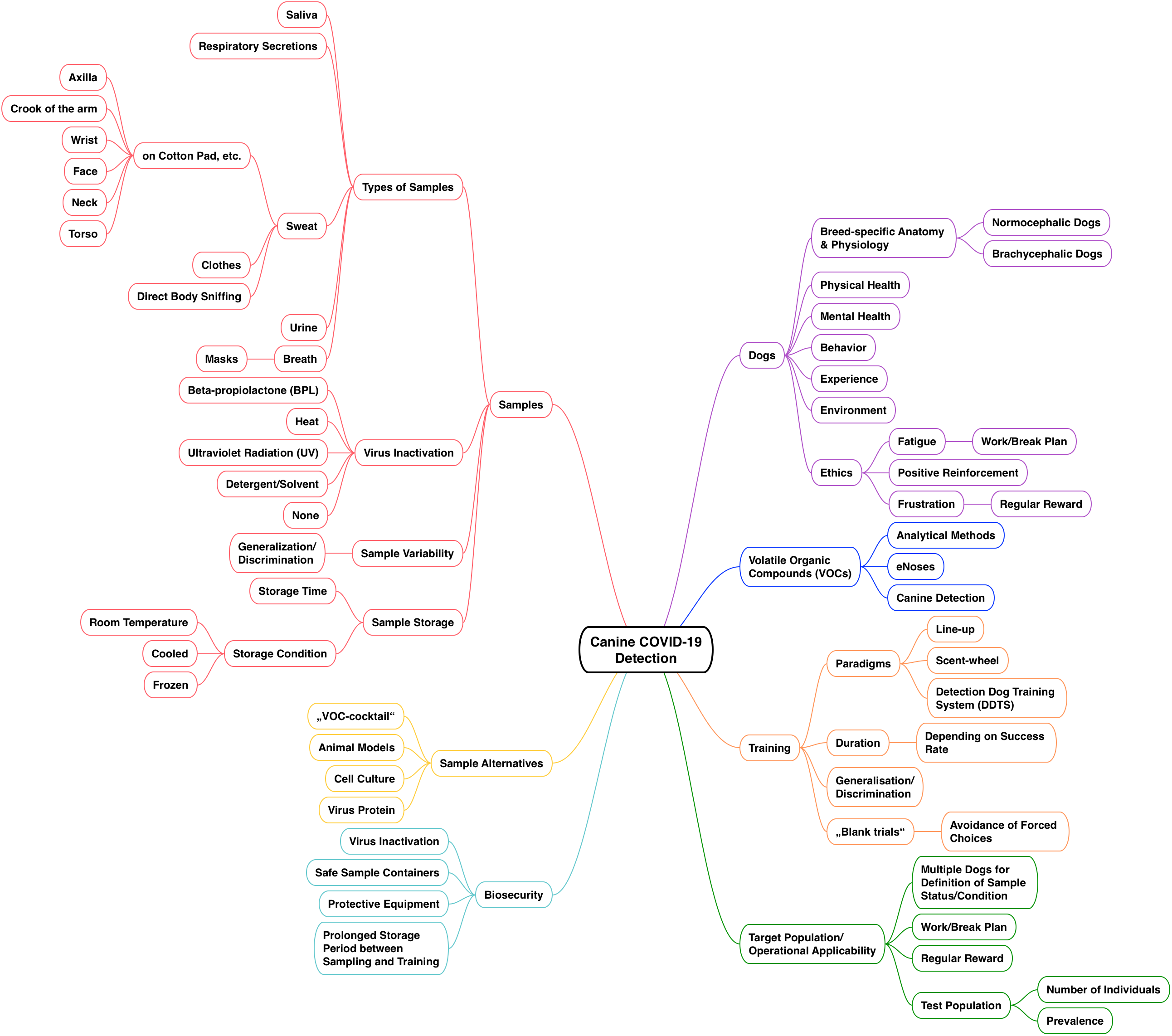

Supplement: Supplementary Figure 1 — Detailed mind map representing areas of interest and related aspects that played a role in the reviewed canine COVID-19-detection studies by Meller et al. (4) and have been highlighted by the experts in this publication. [file Image_1.PNG]
